# A Branched Polyelectrolyte Complex Enables Efficient Flame Retardant and Excellent Robustness for Wood/Polymer Composites

**DOI:** 10.3390/polym12112438

**Published:** 2020-10-22

**Authors:** Yanping Huang, Shuai Zhang, He Chen, Chunxiang Ding, Yan Xuan, Mingzhu Pan, Changtong Mei

**Affiliations:** 1 College of Materials Science and Engineering, Nanjing Forestry University, Nanjing 210037, China; yanpinghuang@njfu.edu.cn (Y.H.); zhangshuai1994@126.com (S.Z.); 13770711109@163.com (H.C.); ding_chunxiang@126.com (C.D.); 2 Advanced Analysis and Testing Center, Nanjing Forestry University, Nanjing 210037, China; xuanyannfu@njfu.edu.cn

**Keywords:** wood/plastic composites, flame retardancy, smoke suppression, toughness, branched polyelectrolyte complex

## Abstract

Wood/thermoplastic composites (WPCs) have been restricted in some fields of building construction and electrical equipment because of their inherent high flammability and lower toughness. In this work, a branched crosslinking network polyelectrolyte complex (PEC) has been designed by incorporation of polyethyleneimine (PEI), a cation polyelectrolyte end capped amine groups, into cellulose nanocrystals (CNC), and ammonium polyphosphate (APP) via self-assembling. The hydrogen bonding interactions, penetration, and mechanical interlock provided by PEC effectively enhance the interfacial bonding within matrix, wood fibers, and flame retardant. Interestingly, it generates abundant micropores on the inner structure of WPC. The excellent interfacial bonding performance and easy-to-move molecular chain successfully transfer the stress and induce energy dissipation, simultaneously giving rise to higher strength and toughness for WPC. As well as the PEC endows WPC with a promotion in both smoke suppression and UL-94 V-0 rate. Additionally, the peak heat release rate and total smoke release for WPC obviously reduce by 36.9% and 50.0% respectively in presence of 25% PEC. A simple, eco-friendly, and concise strategy exhibits prospects for fiber-reinforced polymer composites with effective flame retardancy and mechanical robust properties.

## 1. Introduction

Wood/plastic composites (WPCs) have been widely applied in decking, garden decoration, and packaging materials for their prominent mechanical performance, outstanding water resistance, and environmental friendliness [1]. However, the inherent high flammability of WPC, which has a low limited oxygen index (LOI) of only 19.5–20.2% [2,3], limits its potential applications in decoration, furniture, building construction, and electrical equipment. In the past decades, a great deal of efforts have been paid to promote the flame retardancy and mechanical properties of WPCs simultaneously by incorporation of ammonium polyphosphate (APP) through synergy [4], microencapsulation [5], or surface modification [6]. However, there are still some restrictions in demand of a large amount of organic solvents, and imbibition of toughness and insufficient flame retardancy of WPCs. Hence, a simple, eco-friendly, and concise strategy is needed for higher performance WPCs with the efficient flame retardancy and prominent toughness.

Polyelectrolyte complex (PEC) is a class of soft material condensed from the coacervation of oppositely charged polyelectrolyte solution [7]. By regulating the molecular network between cationic and anionic polyelectrolyte, the PECs can be endowed with biocompatibility, absorbability, and permselectivity, and thus they exhibit attractive applications in catalysis, drug delivery, biosensor, and tissue engineering, etc. Recently, the polyelectrolyte containing P and N elements like chitosan, phytic acid, and APP, has shown great potential in the field of flame retardancy. Li et al. [8] designed a char-forming coating via alternatively depositing poly (allylamine hydrochloride) and sodium hexametaphosphate on cotton fabric, contributing to the self-extinguishing property of cotton fabric. Afterwards, Zhang et al. [9] constructed a green and renewable PEC composed of chitosan and phytic acid for ethylene-vinyl acetate copolymer (EVA). With 20% (mass fraction) PEC, the peak heat release rate of EVA composites showed a 31.1% reduction. Meanwhile, Young’s modulus showed a slight increase, and the excellent ductile nature of EVA was still maintained. Recently, Jing et al. [10] fabricated a core/shell structured PEC named BBH via alternatively assembling polyethyleneimine, phenolic acid, and APP, and applied it to polylactic acid (PLA). With 10% BBH, the treated PLA composites reached UL-94 V-0 rating, and had a 27.3% elongation at break higher than that of neat PLA (8%). Up to now, PEC-based flame additives exhibit synchronous flame retardancy and toughening effect on polymer with active groups, but it is still a challenge for applying PEC in polyethylene, polypropylene materials with low surface energy. In our previous study, an anionic PEC hybrid was established and introduced into WPC with improved flame retardancy and mechanical strengthening [11]. However, the potential of PEC on smoke suppression and toughness remains an attractive challenge. Recently, a branched polymer has been reported which favored the toughness. Compared to other branched polymers, a branched polymer with amine groups also complements the blowing agent for flame retardant during the combustion.

Herein, the branched polyethyleneimine (PEI), a cation polyelectrolyte end-capped amine group, is self-assembled to the anionic polyelectrolytes of APP and cellulose nanocrystal (CNC) to form a branched crosslinking network PEC. The hydrogen bonding interactions, penetration, and mechanical interlock endowed by PEC effectively enhance the interfacial bonding within matrix, wood fiber, and flame retardant. Interestingly, it generates abundant micropores on the inner structure of WPC. It successfully transfers the stress and induces energy dissipation, synchronously giving rise to higher strength and toughness for WPC. As well as the PEC endows WPC with a promotion in both smoke suppression and UL-94 V-0 rate. Compared with our previous study [11], the highest LOI of WPC modified by PEC was increased to 28.7%, and it can pass the UL-94 V-0 combustion test, and TSR decreased from 2380 to 1314 m^2^/m^2^. This novel strategy of PEC expands its value-added fields, such as building construction and electrical equipment for flame-retardant WPC.

## 2. Materials and Methods

### 2.1. Materials

The high-density polyethylene (HDPE) is a homopolymer pellet, grade 5000S, *ρ* = 0.95 g/cm^3^, melt flow index of 0.8~1.2 g/10 min (190 °C/2.16 kg), kindly supplied by Sinopec Yangzi Petrochemical Company Ltd. (Nanjing, China), and crushed into uniform powders within a cyclone crusher. Wood fibers within a size range of 60~80 mesh were collected from the pilot plant of Engineering Research Center of Fast-growing Trees, and they were oven-dried at 103 °C to constant mass. CNC colloids with a solid concentration of 2.2% and a pH value of 6.83 were also provided by the same Research Center. APP (CF-APP II, polymerization degree > 1000) was obtained from Shifang Changfeng Chemical Co, Ltd. (Shifang, China). PEI was kindly supplied by Aladdin Reagent Co., Ltd. (Shanghai, China). All chemical agents were used without further purification.

### 2.2. Fabrication of PEC

**Figure 1:**
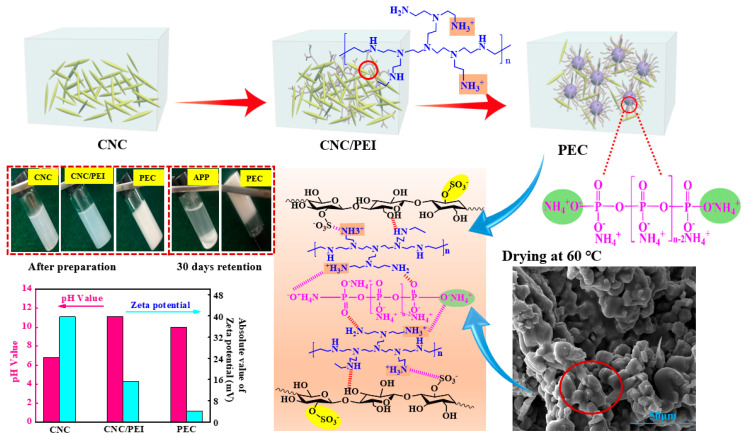
Fabrication schematic diagram of polyelectrolyte complex (PEC) based on cellulose nanocrystals (CNC), polyethyleneimine (PEI), and ammonium polyphosphate (APP), and its storage stability, pH values, and zeta potential values.

Total of 100.0 g CNC colloids with a solid concentration of 2.2% were placed into a 250 mL beaker, and 2.2 g PEI was dispersed in CNC colloids with mechanical stirring at 25 °C for 30 min to get a uniform dispersed CNC/PEI suspension. Afterwards, 24.5 g APP powders were added into the mixed suspension under a continuous mechanical stirring for 10 min. Finally, the mixtures were oven-dried at 50 °C for 2 h until to form gelatinous substances, named as PEC of CNC/PEI/APP. The PEC has a zeta potential value of −4.14 mV, and it can possess outstanding colloidal stability, with no layering phenomenon during retention time within 30 days. A schematic illustration of the PEC fabrication is presented in Figure 1.

### 2.3. Fabrication of WPCs

Flame retardant WPCs were prepared briefly as follows: HDPE powders were successively added into gelatinous PEC with mechanical stirring. The mixtures were then oven-dried at 60 °C for 1 h, subsequently, the treated HDPE and wood fibers were melt compounded at 170 °C for 8 min using a ZG-160 open mill (Dongguan Zhengxin Electromechanical Science and Technology Ltd., Dongguan, China). Afterwards, the blends were compression-molded at 150 °C for 3 min for specimens, and the formulation of WPCs with PEC is shown in Table S1.

### 2.4. Characterization

Zeta potential values were measured by a Malvern Zetasizer Nano ZS (Malvern, UK). Scanning electron microscopy (SEM) (FEI company QUANTA 200) coupled with energy dispersive spectroscopy was used to determine the elemental mapping. The samples were coated with gold before examination. Fourier transform infrared (FTIR) spectra were obtained on a VERTEX 80 infrared spectrum instrument (Bruker, Germany) over a wavenumber of 4000~400 cm^−1^ using KBr pellets. X-ray diffraction (XRD) was performed using an Ultima IV diffractometer (Rigaku, Japan, Cu Kα radiation with λ = 1.5406 Å). X-ray spectra (XPS) was conducted on an AXIS UltraDLD spectroscopy (Shimadzu, Japan). Thermogravimetric analysis (TGA) were performed on a NETZSCH TG 209 F3 at 30~700 °C with a rate of 10 °C/min under N_2_ atmosphere. FTIR was coupled with TGA to investigate the volatile products during thermal degradation at a wavenumber of 4000~400 cm^−1^ with a resolution of 1 cm^−1^. Differential scanning calorimeter (DSC) measurements were performed on a 200 F3 DSC. Dynamic mechanical analysis (DMA) was obtained by Q800 (TA Instruments, Newcastle, WA, USA) in a flexural mode with dual cantilever claws with a rate of 5 °C/min at 1 Hz. Limited oxygen index (LOI) values were tested on an HC-2C oxygen index meter (Jiangning, China) according to ISO 4589. Underwriter Laboratory 94 vertical burning test (UL-94) was conducted on a CZF-2 instrument (Jiangning, China) according to ATSM D 3801. A cone calorimeter test (CCT) was carried out with a cone calorimeter (Fire Testing Technology, East Grinstead, UK) according to ISO 5660. The dimension of the specimen was 100 mm × 100 mm × 4 mm, and the external heat flux of 50 kW/m^2^ was applied. Tensile properties were measured with an electronic versatile testing machine (CMT6104, Shenzhen Xinsansi Material Testing Co, Ltd., Shenzhen, China). The unnotched Charpy impact test was performed using a SANS ZBC 1251-1 tester.

## 3. Results

### 3.1. Characterization of PEC

**Figure 2:**
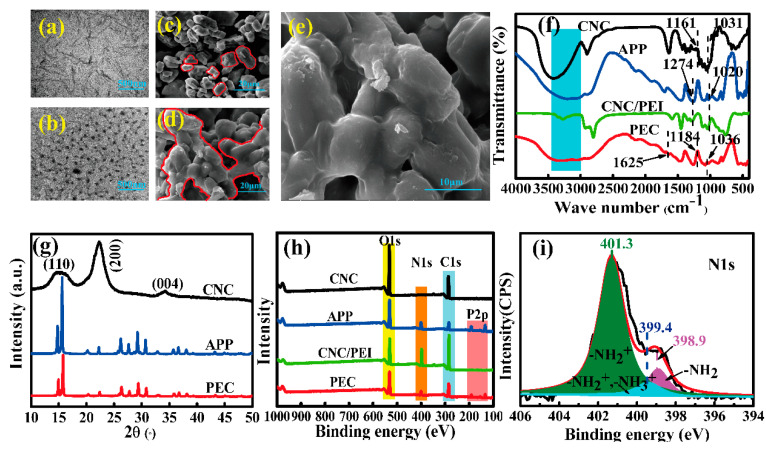
Morphological and physicochemical characterization of PEC. TEM of CNC (**a**) and APP (**b**), SEM of APP (**c**), PEC (2000×, **d**), and PEC (4000×, **e**), FTIR (**f**), XRD (**g**), XPS (**h**) spectra of PEC, and its N1s high-resolution spectra of PEC (**i**).

Figure 2a shows the TEM morphology of CNC, a rod-like structure with a diameter of 20~55 nm and a length of 210~520 nm in our previous report [11]. In addition, it has a large surface area (150~500 m^2^·g^−1^), which makes it easy to disperse in water to form a chiral nematic structure [12]. APP displays spherulitic particles (Figure 2b), and it can also be observed from SEM image that the APP is composed of small irregular particles with a smooth surface and no obvious cracks (Figure 2c). With successively self-assembling CNC and PEI, APP particles are coated with a thick layer coating, and they stick to each other, forming an integrated system (Figure 2d,e). For APP (Figure 2f), the bands at 3400~3000 cm^−1^ are assigned to the asymmetrical stretching vibration of NH_4_^+^ [13]. The peaks at 1274 and 1020 cm^−1^ are assigned to the absorption vibration of P=O and stretching vibration of P–O, respectively [14]. For PEC, the peak at 1184 cm^−1^ assigns to C–O–C asymmetric stretching at β-glucosidic linkage, 1036 cm^−1^ ascribes to C–O at C–6 stretching [15], and the peak at 1625 cm^−1^ arises from the bending vibration of –NH [16], which indicates that CNC and PEI are successfully introduced into APP surface. The peaks at 2θ = 14.8° and 15.6° are both present in APP and PEC (Figure 2g), indicating PEC remains the crystalline structure of APP II [14]. To clarify the surface chemical structure of PEC, XPS measurement was subsequently conducted to evaluate the content and distribution of the element. The peaks at 133, 168, 285, 401, and 531 eV (Figure 2h) are assigned to P2p, S2p, C1s, N1s, and O1s, which further verify the presence of CNC and PEI within APP molecules. High-resolution of N1s spectra was conducted to study the chemical binding within PEC (Figure 2i). New peak at 399.4 eV assigns to –NH^2+^ and –NH^3+^ [17], the peak at 398.9 eV attributes to amine groups, and 401.3 eV assigns to protonated amine groups [18]. It is noted that CNC exhibits a strong anion polyelectrolyte with a higher absolute zeta potential value of −39.70 mV due to the surface grafting of sulfonic acid group with negative charge. APP is a kind of weak anionic polyelectrolyte with a zeta potential value of −1.15 mV, and it can partially dissociate into NH^4+^ groups and negatively charged polyphosphates. Meanwhile, alkaline imide groups on PEI is deionized to release OH– and –NH^3+^ groups. Consequently, a comprehensive ion complexation is ultimately formed between the positively charged –NH^3+^ groups from PEI and the negatively charged polyphosphate chain and sulfonic acid groups from CNC molecules. Moreover, hydrogen bonding (–NH·····O) and (–N·····OH) between CNC, PEI, and APP also strengthens the crosslinked network of PEC, which is shown in Figure 1, and it is crucial for the forthcoming interfacial regulation and char formation.

### 3.2. Flame Retardancy of WPC/PEC Composites

The LOI, UL-94, and CCT were performed to evaluate the flame retardancy of WPC with PEC, and the detailed results are illustrated in Table 1. Neat WPC is an easily flammable material with LOI of only 19.8%, and it does not pass the UL-94 rating. For WPC/APP 15%, the LOI value increases to 23.9%, but it still fails to pass the UL-94 rating. For WPC/PEC 15%, its LOI value is increased to 24.4%. Obviously, PEC can improve the LOI of WPC more effectively than APP. Moreover, increasing PEC to 25% reaches a higher LOI of 28.7% and it obtains a UL-94 V-0 rating for WPC. All samples burn out and the burned parts are covered with intumescent chars. Among them, neat WPC is almost completely burned, and incorporating APP slightly improves the combustion characteristics. Interestingly, adding PEC significantly reduces the combustion portion of WPC, accounting for less than half of the entire sample. The combustion parts of WPC/PEC 20% and WPC/PEC 25% reduced accordingly. The combustion of samples after the UL-94 test is illustrated in Figure S1 (in the Supplementary Materials).

**Figure 3:**
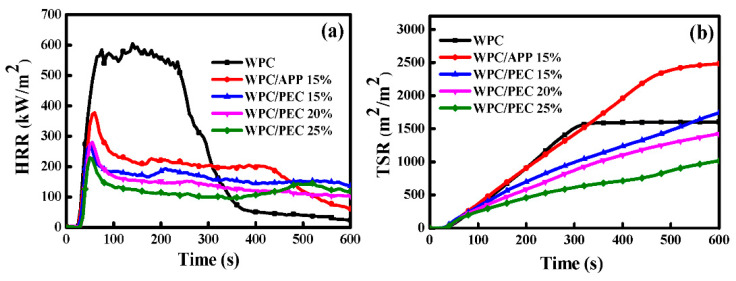
CCT results of WPC with PEC: (**a**) HRR and (**b**) TSR.

In terms of the CCT results, the addition of APP and PEC both decrease the HRR of WPC during the whole combustion (Figure 3a). For WPC/APP 15%, the average and peak HRR decrease from 261.56 and 621.58 kW/m^2^ to 109.55 and 373.92 kW/m^2^, respectively, which are 58% and 40% lower than that of neat WPC. For WPC/PEC 15%, the average and peak HRR decrease by 61% and 55%, respectively, which are greater than WPC/APP 15%. Especially for WPC/PEC 25%, the average and peak HRR mostly decrease by 68% and 62% lower than that of neat WPC. Interestingly, adding PEC causes a prominent decrease in the TSR of WPC compared to that of APP (Figure 3b). It is noted that the TSR of WPC/APP 15% is relatively high, which is 2626.1 m^2^/m^2^. That is mainly due to the incomplete combustion by intumescent carbon layer carbonized with APP, which is a commonly mentioned phenomenon [13,19]. Interestingly, PEC sharply decreases the TSR to 1897.8 m^2^/m^2^ for WPC, corresponding to a 27.7% reduction compared to that of APP. With increasing PEC to 25%, the TSR eventually is decreased to 1313.78 m^2^/m^2^, which is even lower than that of neat WPC. Meanwhile, the results of SEA are consistent with TSR, further proving the lower smoke emission of WPC with PEC than APP.

### 3.3. Flame Retardancy Mechanism

**Figure 4:**
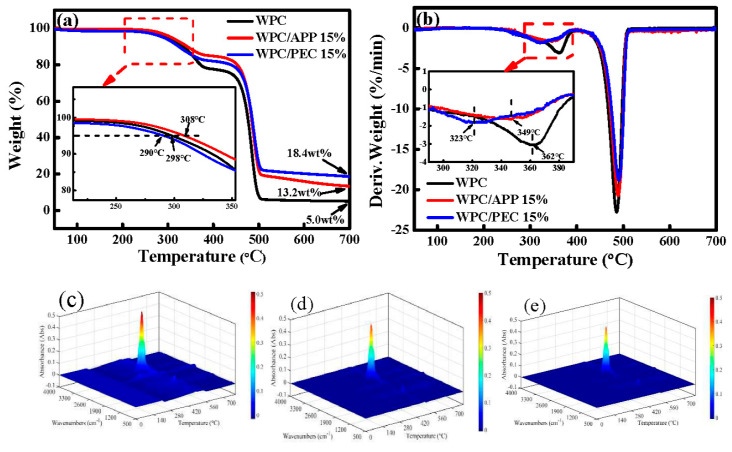
Thermal results of WPC with APP and PEC: TG (**a**) and DTG (**b**) curves, and the 3D-FTIR spectra of the volatile pyrolysis products from room temperature to 700 °C during the whole pyrolysis: WPC (**c**), WPC/APP 15% (**d**), WPC/PEC 15% (**e**).

Figure 4a,b shows the thermally decomposing behaviors of WPC with 15% APP and PEC. The neat WPC starts to thermally decompose at 298 °C (*T*_5%_, the temperature at 5% weight loss). Moreover, it has two peak temperatures of thermal decomposition (*T*_peak_) at 362 and 486 °C, respectively, corresponding to the severe thermal degradation of wood fibers and HDPE molecules [4,20]. For WPC/APP 15%, *T*_5%_ is shifted to a higher temperature of 308 °C. The char residue is significantly increased to 13.2 wt.%. For WPC/PEC 15%, its *T*_5%_ is shifted to a lower temperature of 290 °C assigning to the earlier decomposition of PEC induced by catalysis and dehydration of CNC [11], which is beneficial for the char forming. Meanwhile, *T*_peak_ (491 °C) of WPC/PEC 15% is slightly higher than that of neat WPC and WPC/APP 15%. Moreover, its char residues at 700 °C remains 18.4 wt.%, inherently possesses more thermal stability at higher temperatures.

Figure 4c–e shows 3D images of the FTIR spectra of total gaseous products during the whole pyrolysis. For neat WPC, it is clear that almost no gas product is released below 280 °C. WPC starts to decompose at 298 °C, the peak at 2100~2200 cm^−1^ is attributed to CO [21]. As time continues, a large number of gaseous compounds, such as CO_2_, H_2_O, and some alkanes, ethers, phenols, ketones, aldehydes, and other organic substances begin to volatilize. For WPC/APP 15%, it starts to decompose at 308 °C, the products evolved are confirmed by the characteristic peaks of CO (2200~2300 cm^−1^), NH_3_ (3000~3200 cm^−1^, 800~1000 cm^−1^), H_2_O (3500~4000 cm^−1^), and phosphorus oxides (1200~1300 cm^−1^) [22]. For WPC/PEC 15%, the pyrolysis is prior to a lower temperature of 290 °C, and it shows similar peaks on the TG-FTIR spectrum compared to that of WPC/APP 15%. However, the corresponding peaks present in WPC/PEC 15% are weaker than that of WPC/APP 15%, indicating that combustion process of WPC/PEC 15% is relatively gentler and releases less gaseous compounds, resulting in a higher char residue at 700 °C, which further illustrates that PEC can improve the smoke suppression of WPC.

**Figure 5:**
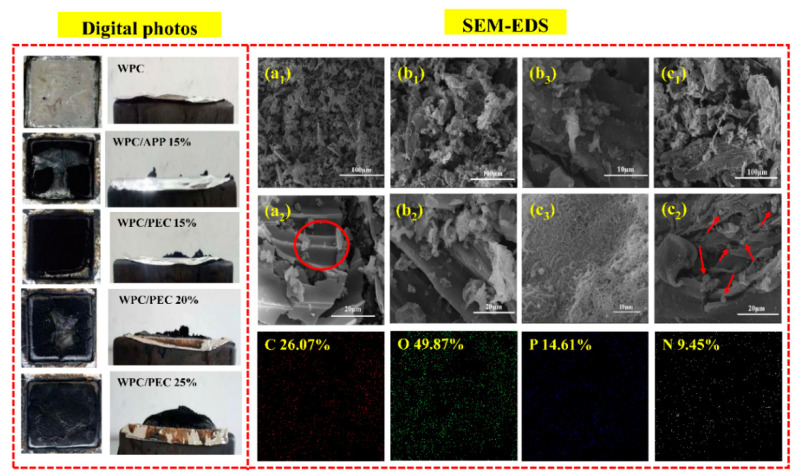
Digital photos of char residues from CCT, and their SEM images of WPC (**a_1_**,**a_2_**), WPC/APP 15% (**b_1_**,**b_2_**,**b_3_**), and WPC/PEC 15% (**c_1_,c_2_,c_3_**) and its EDX pattern of char residues.

Figure 5 shows the digital and microscopic morphology of the char layer of WPC with PEC after CCT. For neat WPC, only a tiny granular char layer is present on the surface of the tin foil. For WPC/APP 15%, it exhibits a thinner, uncontinuous sheet-like structure with some detectable cracks. For WPC/PEC 15%, a thick and continuous char layer with an increased physical integrity appears. With increasing PEC to 20% and 25%, the char layer is more and more obviously observed. To further investigate the microscopic structure, char residues were further conducted with SEM. For neat WPC, it can be observed in Figure 5(a_1_,a_2_) that the char residue displays a loose surface with many cracks, and this phenomenon has also been reported by Liu et al. [23]. For WPC/APP 15%, incorporating APP results in the formation of a more compact char layer and the disappearance of huge cracks (Figure 5(b_1_,b_2_)). Also, there are still many channels and holes on the surface, and this type of residual char cannot serve as a suitable thermal insulator layer [24]. Moreover, there is no obvious adhesion between carbon skeleton carbonized from wood fibers and other residues, and they cannot support a continuous and integrated network, which results in undesirable flame retardancy. For WPC/PEC 15%, incorporating PEC makes the residual char layer denser and more stable (Figure 5(c_1_,c_2_)). Moreover, the abundance of micropores formed within char layer structure (Figure 5(c_3_)), leads to the formation of intumescent char layer. It can be clearly seen that the better adhesion between carbon skeleton and the substrate leads to a more uniform dispersion of the residues, thereby further effectively strengthening the char layer influenced by PEC than APP. Additionally, many spherical protrusions appear on the surface of the carbon skeleton (Figure 5(c_2_)). It may be attributed to the presence of N-contained substrates during the pyrolysis of PEC. The EDS analysis further confirms that higher amounts of C and N are present for WPC with PEC than APP (Figure S2 in the Supplementary Materials), which are beneficial to the formation of the intumescent char layer.

**Figure 6:**
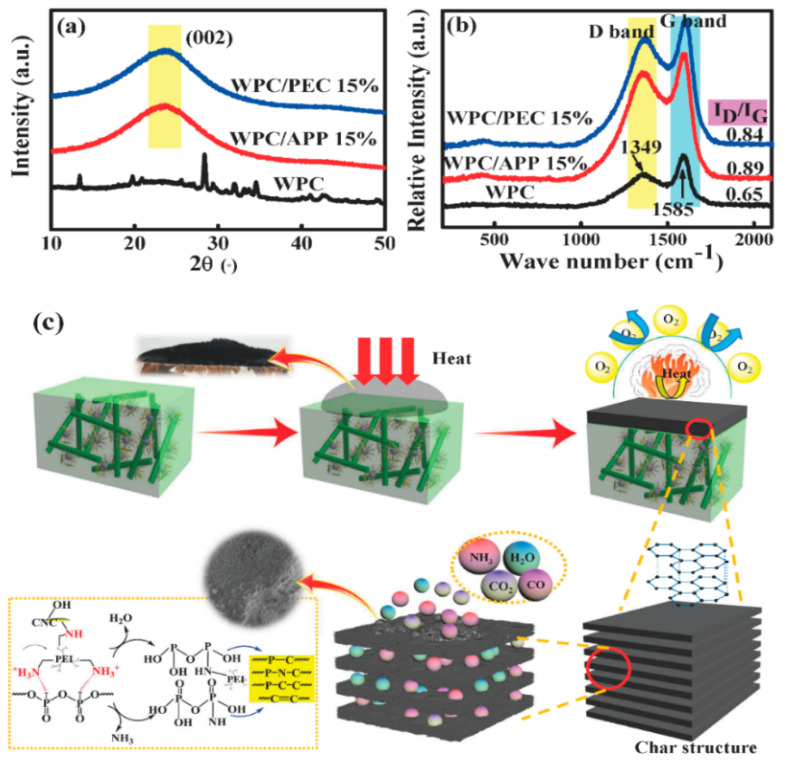
Chemical structure of char residues: XRD (**a**) and Raman spectra (**b**), and possible flame retardancy mechanism of WPC with PEC (**c**).

Figure 6a shows the XRD spectra of the char residues after CCT. For WPC/APP 15%, the diffraction pattern reveals a crystalline phase of a phosphorus-based residue. A new diffraction peak appearing at 2θ value of 25° ascribes to the reflection from the (002) lattice plane [4,25]. For WPC/PEC 15%, the XRD spectra does not change significantly. Figure 6b further shows Raman spectra of the char residues. Two main bands obviously appear at 1585 cm^−1^ (G band) and 1349 cm^−1^ (D band). The char residues show a high I_D_/I_G_ value of 0.89 for WPC/APP 15%, indicating a low graphitization degree. While its I_D_/I_G_ value decreases to 0.84 for WPC/PEC 15%, revealing an increased graphitization degree.

According to the above analysis, the flame retardancy mechanism of WPC/PEC is as follows. During the combustion process, incorporating PEC shifts the pyrolysis of WPC to a lower temperature of 290 °C induced by the catalysis and dehydration of CNC with abundant sulfonate groups present on its surface [26], leading to an earlier formation of the char layer and an effusion of the degraded volatiles. The degraded volatiles released by the early pyrolysis of composite materials are NH_3_, H_2_O, CO, and CO_2_; on the one hand, the oxygen content around the bulk material is diluted so as to inhibit the flame combustion and achieve flame retardant effect in the gas phase; on the other hand, because of the gas swelling, the expanded char layer is gradually formed so as to isolate the heat and oxygen (Figure 5(c_1_)). Meanwhile, the highly crosslinked polyphosphate chain in PEC releases phosphoric acid and phosphorus-oxygen species, which can further accelerate the dehydration and esterification reactions of CNC and wood fibers, and it effectively promote the graphitization of char layer. Besides, the improved interfacial compatibility, which is deeply discussed in the forthcoming mechanical properties, enhances the adhesion between carbon skeleton and the substrate, so that a more continuous, denser, and higher graphitization intumescent carbon layer is gradually formed, which inhibits the wick effect of wood fibers and blocks the heat transfer and the flow of combustible gas, that plays a remarkable flame retardant effect on a condensed phase [6]. Furthermore, a large number of micropores within intumescent char structure (Figure 5(c_3_)) tend to absorb smoke released during combustion process owing to their small particle size and large specific surface area, further achieving a smoke suppression effect, resulting in a prominent reduction in TSR and SEA, and the possible reaction of PEC within WPC is illustrated in Figure 6c. As a result, PEC has an effective flame retardant effect on WPC in both the gas phase and the condensed phase.

### 3.4. Mechanical Properties of WPC/PEC Composites

**Figure 7:**
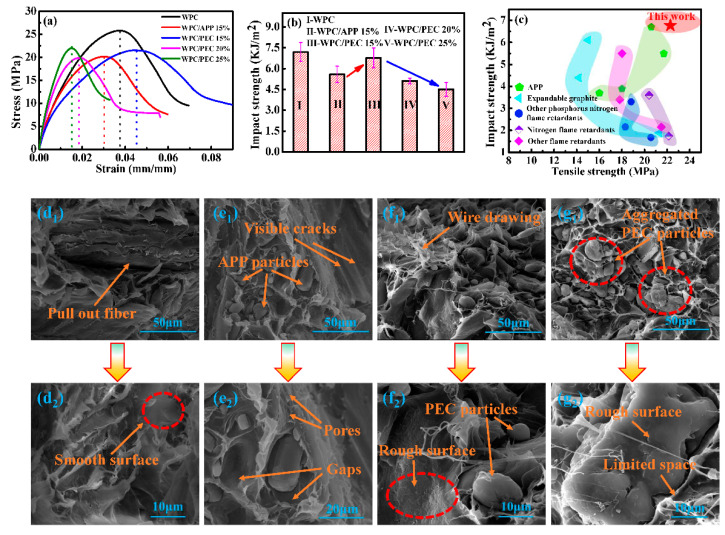
Mechanical properties and fractural morphology of WPC with APP and PEC: (**a**) Strain–stress curves, (**b**) impact strength, and (**c**) comparison with other reported flame retardants [2,26,27,28,29,30,31,32]. SEM images of WPC (**d_1_**,**d_2_**), WPC/APP 15% (**e_1_**,**e_2_**), WPC/PEC 15% (**f_1_**,**f_2_**), and WPC/PEC 25% (**g_1_**,**g_2_**).

Figure 7 shows the mechanical properties of WPC with PEC, and gives a comparison between PEC and other flame retardants for WPC. It is noted that neat APP significantly deteriorates the strength and toughness of WPC due to the weak interfacial compatibility. Accordingly, the tensile and impact strength decrease to 20.55 MPa and 5.58 kJ/m^2^, respectively for WPC with 15% APP (Figure 7a,b). Interestingly, PEC obviously improves the mechanical performances of WPC compared with the relevant equal of APP. The tensile and impact strength significantly increases to 22.28 MPa and 6.77 kJ/m^2^ for WPC with 15% PEC. As compared to the mechanical properties of some other flame retardant-WPC [2,27,28,29,30,31,32,33] (Figure 7c), such as WPC with expandable graphite, APP, and synergist, even some other phosphorus nitrogen flame retardants, they generally have a lower impact (1.9~6.7 kJ/m^2^) or tensile strength (10.4~22.1 MPa), which cannot take into account both, so the overall mechanical properties are not ideal. On the contrary, PEC obviously generates a synchronous promotion on strengthening and toughening WPC. In addition, compared with our previous work [11], the maximum tensile elongation at break is increased from 5.9% to 8.4% in this work, and the tensile strength remains unchanged. With increasing PEC addition, the strength has been further improved, and the interfacial improvement effect becomes stabilizing with the addition of PEC reached to 20%, as the tensile strength remained ca. 23 MPa and is basically unchanged even the addition of PEC is up to 25%. However, its toughness has shown a downward trend for WPC with 20%, and 25% PEC.

The dynamic mechanical properties of WPC with PEC is also presented in Figure S3a,b (in the Supplementary Materials). Adding APP increases the storage modulus (E′) to 2622 from 2158 MPa of neat WPC. According to the glass transition temperature (*T*_g_), APP slightly decreases the *T*_g_ to 53.56 °C from 54.56 °C for neat WPC due to the weak interfacial compatibility. Comparatively, incorporating PEC further exhibits the reinforcement effect on the E′ of WPC. Interestingly, the *T*_g_ is transferred to a lower temperature of 52.91 °C for WPC with PEC compared to APP. Subsequently, the *T*_g_ once again shifts to high temperatures with the increase in PEC to 20%, and 25%. Figure S3c,d further displays the crystallization behavior of WPC with PEC. Incorporating 15% PEC abruptly decreases the melting enthalpy (Δ*H*_m_) for WPC compared to APP. Moreover, the crystallinity (*X*_c_) is also reduced by 29.6% for WPC. In contrast, the *X*_c_ of WPC/PEC 25% has been greatly improved, which is 36.5% higher than that of WPC/PEC 15%.

Figure 7d–g shows the morphologies of the fracture surfaces of WPC with PEC. For neat WPC (Figure 7(d_1_,d_2_)), the fracture surface between the wood fibers and HDPE is distinct and smoother, and the wood fibers exhibit obvious pull-out from HDPE matrix contributed to the weak interfacial compatibility. For WPC/APP 15% (Figure 7(e_1_,e_2_)), many pores are generated within the structure of composites, wherein, APP particles are randomly distributed in these pores, and the interfacial gaps are obviously appeared between the wood fibers, APP and HDPE matrix. Besides, the agglomerated APP particles could be a site of stress concentration, which can act as a micro-crack initiator that accelerated crack growth thereby decreasing the strength and toughness of WPC [5]. Interestingly, for WPC/PEC 15%, the pull-out of wood fibers from the HDPE matrix disappears and there are some obvious wire drawing on the surface of HDPE (Figure 7(f_1_,f_2_)). PEC is spherical-like and evenly embedded in the matrix. Moreover, FTIR shows a red shift of hydroxyl groups transferring from 3345 to 3280 cm^−1^ for WPC with PEC from 15% to 25% (Figure S4 in the Supplementary Materials), indicating a formation of hydrogen bonding between amine and hydroxyl group from PEC and wood fibers, successively, it improves interfacial bonding between flame retardant and wood fibers. With increasing PEC to 20% (Figure S5 in the Supplementary Materials) and 25% PEC (Figure 7(g_1_,g_2_)), the agglomeration of PEC gradually appears within the composite structure, leading to the disappearances of abundant micropores. Also, the agglomerated PEC displays irregularly on the exterior surface.

**Figure 8:**
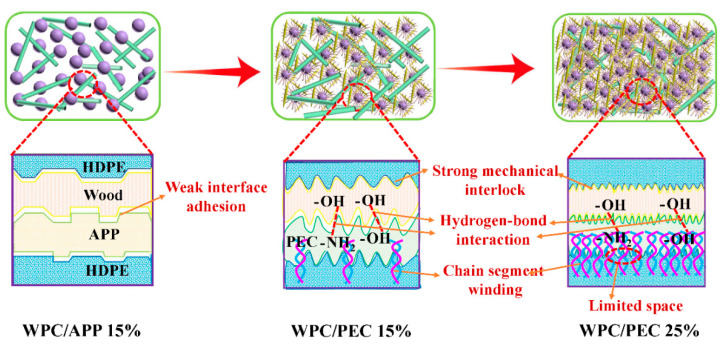
PEC distribution within WPC and possible interfacial bonding mechanisms.

Combining the above-mentioned results, the hydrogen bonding forms between the PEC and wood fibers during the fabrication of WPC with PEC. As well as, the organic side chains on the PEI increase the compatibility between PEC and the HDPE matrix, and greatly contribute to the enhancement of dissolution and penetration. Parallel to this, the PEC surface becomes rough after self-assembling CNC and PEI (Figure 2d,e), thus the mechanical interlock becomes stronger for WPC/PEC than WPC/APP. Hence, such “soy-bean” PEC particles are evenly distributed within composites (Figure 7(f_1_,f_2_)). During stretching, the stress transfers effectively from the HDPE matrix to wood fibers through the enhanced interface via hydrogen bonding interactions, penetration, and mechanical interlock. Additionally, appropriate CNC and PEI within PEC networks provide sufficient micropores, tending to provide the deformation space. When WPC is under shock, its chain segment is easier to deform and absorb energy [34]. Consequently, the excellent interfacial bonding performance and easy-to-move molecular chain endowed by PEC make WPC possess higher strength and toughness simultaneously compared to that of APP. With excessive PEC (20% and 25%), the agglomeration of PEC gradually induces the disappearance of abundant micropores (Figure S5 and Figure 7(g_1_,g_2_)), and it restricts the chain segment motion of HDPE and PEI, thereby hindering the energy absorption, resulting in a reduction in toughness. However, the interfacial compatibility provided by PEC dominates stress transformation from HDPE matrix to wood fibers, leading to a rise in strength without falling. The possible strain transformation and absorb energy mechanism is illustrated in Figure 8.

## 4. Conclusions

To prepare a high-performance WPC with high flame retardancy and mechanical robustness, a branched crosslinking network PEC based on CNC, PEI, and APP has been self-assembled via ion complexation and hydrogen bonding in this work. Incorporating PEC effectively promotes the flame retardancy of WPC greater than APP does. The limiting oxygen index reaches 28.7%, and the UL-94 test can pass the V-0 rating for WPC with 25% PEC. The flame retardant mechanism of WPC/PEC composites is also deeply discussed. Accordingly, the peak HRR and TSR for WPC significantly reduced by 36.9% and 50.0% respectively in presence of 25% PEC. Meanwhile, the better interfacial compatibility effectively transfers stress to wood fibers, and the abundant micropores within composite structure partially induce energy dissipation available, which make the WPC with the same amount of PEC have higher strength and toughness simultaneously than APP. Overall, the demonstrated PEC shows great prospect as an effective flame retardant and mechanical properties modifier for WPC.

## Figures and Tables

**Table 1:** Flammability results for WPC with PEC obtained from limited oxygen index (LOI), UL-94, and from cone calorimeter test (CCT) *.

Samples	LOI (%)	UL-94 (3.2 mm)		Average HRR	Peak HRR	THR	TTI	SEA	TSR	MLR
		Rating	Dripping	(kW/m^2^)	(kW/m^2^)	(MJ/m^2^)	(s)	(m^2^/kg)	(m^2^/m^2^)	(mg/s)
WPC	19.8 (0.15)	NR	No	261.56 (15.5)	621.58 (23.0)	147.7 (5.0)	20.5 (0.7)	330.57 (89.1)	1381.9 (308.3)	63.6 (0.8)
WPC/APP 15%	23.9 (0.37)	NR	No	109.55 (0.6)	373.92 (5.2)	128.7 (0.7)	22.5 (0.7)	616.93 (159.2)	2626.1 (469.0)	32.3 (2.6)
WPC/PEC 15%	24.4 (0.17)	NR	No	102.11 (3.6)	279.68 (11.9)	119.9 (4.2)	22.5 (0.7)	470.20 (54.6)	1897.8 (209.2)	30.4 (0.005)
WPC/PEC 20%	26.6 (0.15)	V-1	No	81.89 (8.6)	283.90 (4.9)	145.2 (15.5)	27.0 (4.2)	448.61 (0.8)	2323.5 (289.8)	25.75 (3.1)
WPC/PEC 25%	28.7 (0.21)	V-0	No	84.11 (0.1)	235.85 (9.8)	148.9 (0.2)	28.5 (0.7)	256.85 (57.6)	1313.78 (238.5)	25.55 (1.1)

* HRR: heat release rate, THR: total heat release, TTI: time to ignite, SEA: specific extinction area, TSR: total smoke release, MLR: mass loss rate.

## Supplementary Materials

The following are available online at https://www.mdpi.com/2073-4360/12/11/2438/s1, Figure S1: Digital photos of (a) WPC, (b) WPC/APP 15%, (c) WPC/PEC 15%, (d) WPC/PEC 20%, and (e) WPC/PEC 25% from vertical burning test (UL-94). Table S1: Formulation of WPCs with and APP and PECs (wt.%). Figure S2: EDX pattern of the char residues from CCT: (a) WPC and (b) WPC/APP 15%. The scale bar of SEM was 10 μm. Figure S3: Thermomechanical results: DMA curves of WPC with APP and PEC. (a) storage modulus (E′) and (b) loss modulus (E″). DSC curves of WPC with 15% APP and PEC. (c) cooling and (d) second heating. Figure S4: FTIR spectra of WPC/PEC composites. Figure S5: Fractural SEM images of WPC/PEC 20%.
