# Supplementary material for: A Branched Polyelectrolyte Complex Enables Efficient Flame Retardant and Excellent Robustness for Wood/Polymer Composites
Source: Polymers (Basel). 2020 Oct 22;12(11):2438. doi: 10.3390/polym12112438 (PMC7770577; doi:10.3390/polym12112438)
Supplement: Supplementary file 1 [file polymers-12-02438-s001.pdf]

polymers

Supplementary Materials

A Branched Polyelectrolyte Complex Enables Efficient Flame Retardant and Excellent Robustness for Wood/Polymer Composites

Yanping Huang[1], Shuai Zhang[1], He Chen[1], Chunxiang Ding[1], Yan Xuan[2], Mingzhu Pan[1*] and Changtong Mei[1*]

[1] College of Materials Science and Engineering, Nanjing Forestry University, Nanjing 210037, China; yanpinghuang@njfu.edu.cn (Y.H.); zhangshuai1994@126.com (S.Z.); 13770711109@163.com (H.C.); ding_chunxiang@126.com (C.D.)

[2] Advanced Analysis and Testing Center, Nanjing Forestry University, Nanjing 210037, China; xuanyannfu@njfu.edu.cn (Y.X.)

* Correspondence: mzpan@njfu.edu.cn (M.P.); mei@njfu.edu.cn (C.M.)

Table S1. Formulation of WPCs with and APP and PECs (wt%).

Samples	HDPE	Wood fibers	Flame retardants
WPC	70	30	0
WPC/APP 15%	55	30	15
WPC/PEC 15%	55	30	15
WPC/PEC 20%	50	30	20
WPC/PEC 25%	45	30	25

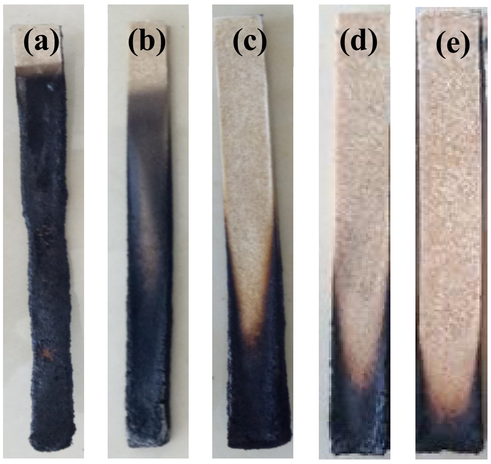

Figure S1. Digital photos of (**a**) WPC, (**b**) WPC/APP 15%, (**c**) WPC/PEC 15%, (**d**) WPC/PEC 20%, and (**e**) WPC/PEC 25% from vertical burning test (UL-94).

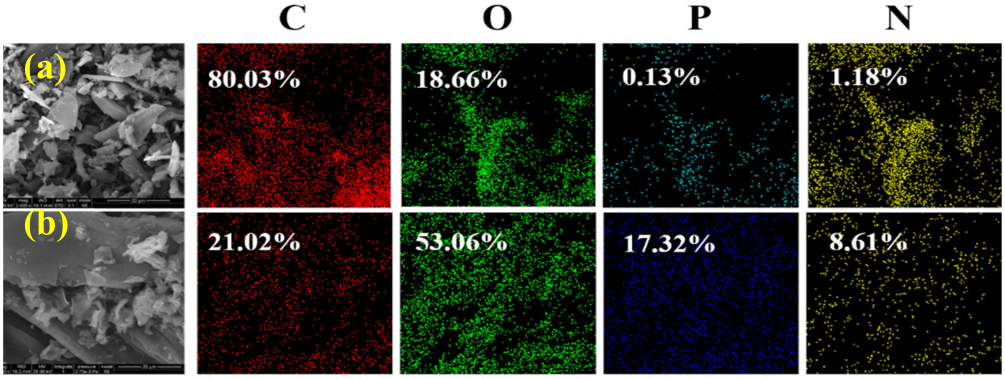

Figure S2. EDX pattern of the char residues from CCT: (**a**) WPC and (**b**) WPC/APP 15%. The scale bar of SEM was 10μm.

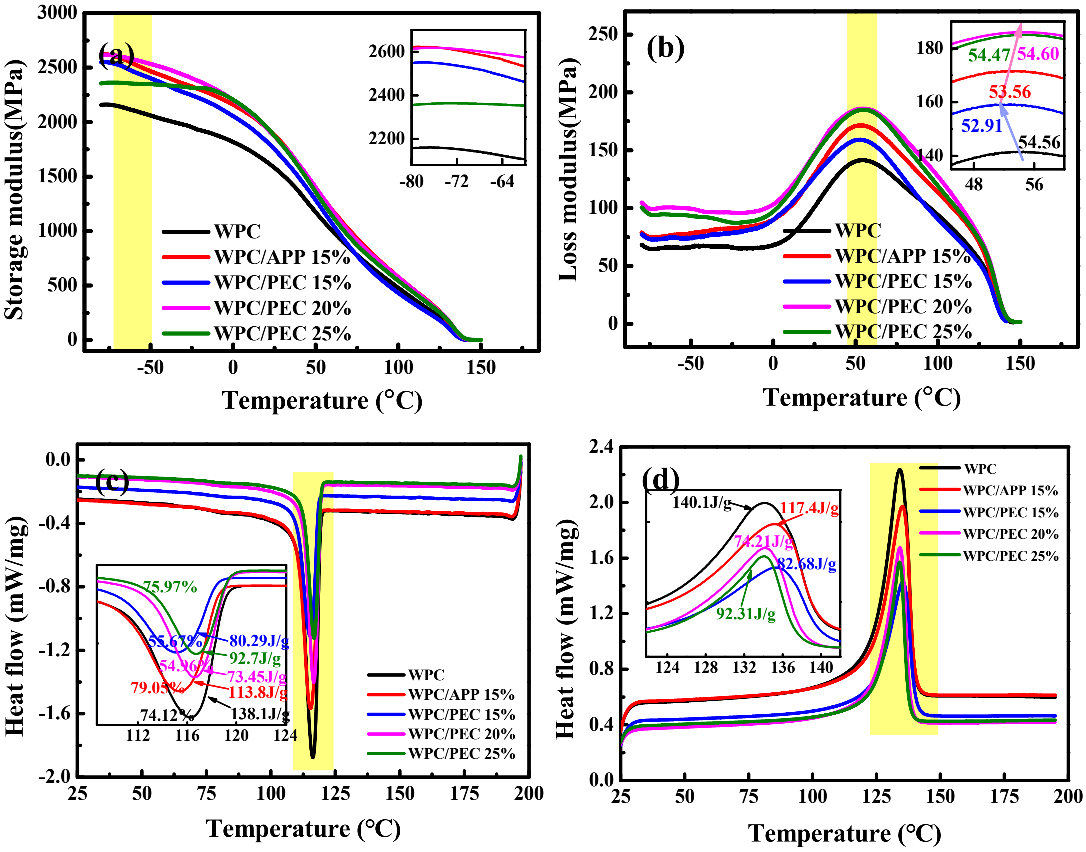

Figure S3. Thermomechanical results: DMA curves of WPC with APP and PEC. (**a**) storage modulus (E') and (**b**) loss modulus (E''). DSC curves of WPC with 15% APP and PEC. (**c**) cooling and (**d**) second heating.

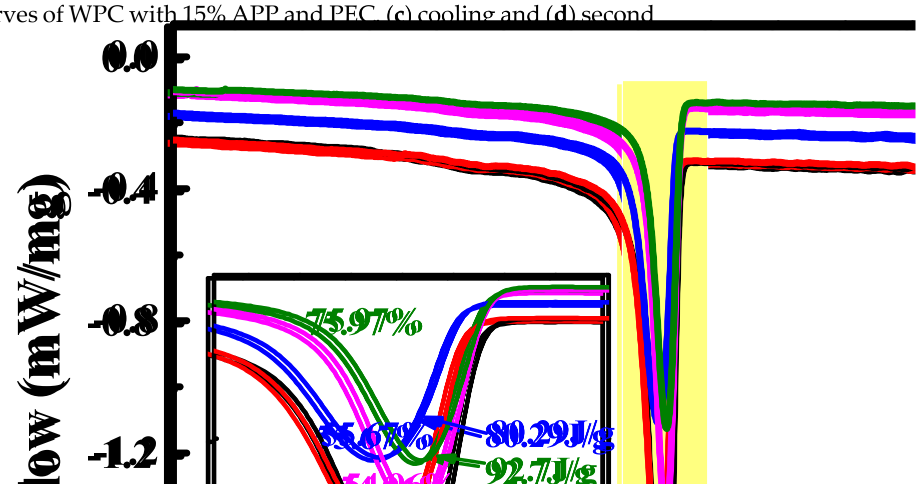

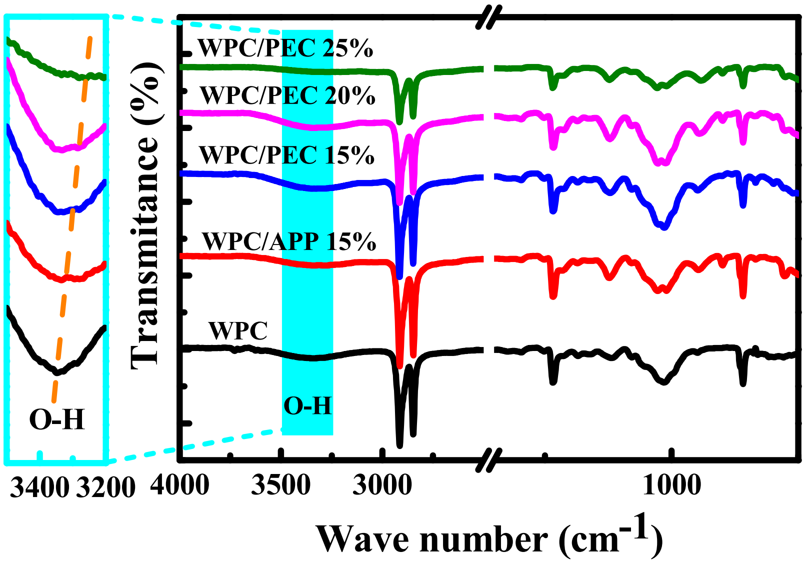

Figure S4. FTIR spectra of WPC/PEC composites.

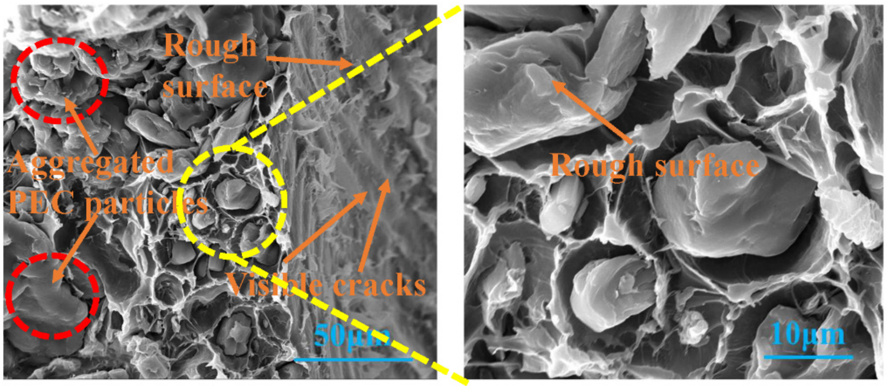

Figure S5. Fractural SEM images of WPC/PEC 20%.

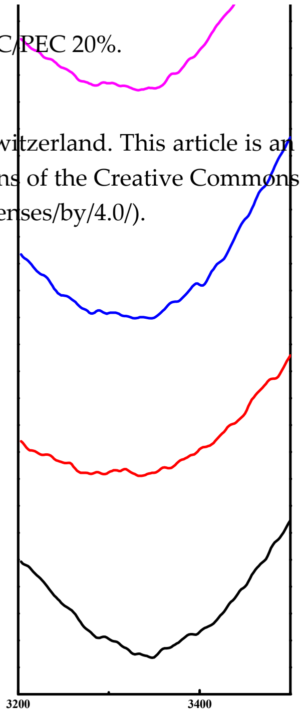

© 2020 by the authors. Licensee MDPI, Basel, Switzerland. This article is an open access article distributed under the terms and conditions of the Creative Commons Attribution (CC BY) license (http://creativecommons.org/licenses/by/4.0/).
